# Individual Characteristics Associated with Fears and Prevention Behaviors Related to Respiratory Infectious Disease among South Korean Adults Using Complex Sample Design

**DOI:** 10.3390/healthcare12191924

**Published:** 2024-09-25

**Authors:** Gunsoo Han, Jae-Ahm Park

**Affiliations:** Department of Sports and Leisure Studies, College of Humanities, Daegu University, 201 Daegudae-ro, Gyeongsan-si 38453, Republic of Korea; gunsoo@gmail.com

**Keywords:** individual characteristics, physical activity, self-perceived health status, COVID-19, fear

## Abstract

Objectives: This study aimed to identify the relationships among individual characteristics, COVID-19-related fears, and infection-prevention behaviors using data from 228,932 adults (weighted to 43,583,798.71) aged 19 and older who participated in the 2021 Community Health Survey conducted by the Korea Disease Control and Prevention Agency. Methods: The complex sample design analysis using SPSS 20.0 revealed the following. Results: Women had statistically significantly higher COVID-19-related fears and a higher level of preventive behaviors compared to men. During the COVID-19 pandemic, the level of physical activity among men was higher than that of women. Compared to the period before COVID-19, the frequency of smoking and drinking decreased for both men and women, with the reduction being more pronounced in men than in women. The high-exercise group had a lower level of COVID-19-related fears. Self-perceived health status was inversely and significantly related to COVID-19-related fears. The low-exercise group had a lower score for self-perceived health status than the high-exercise group. Conclusions: This study demonstrated that maintaining sufficient physical activity positively influences self-perceived health and reduces anxieties related to COVID-19 infection and fatalities. The results highlight the importance of physical activity and the need to identify strategies that effectively maintain regular exercise while adhering to infection-prevention measures during pandemics.

## 1. Introduction

### 1.1. Needs of the Study

In March 2020, COVID-19 was declared a global pandemic by the World Health Organization, and as of November 2023, over 771 million cases and nearly 7 million fatalities have been officially recorded [1,2]. The pandemic profoundly disrupted everyday life and precipitated a significant increase in mental health issues [3,4]. A study involving 113,285 individuals revealed that within the first seven months of the pandemic, the prevalence of depression, anxiety, and stress reached 20%, 35%, and 53%, respectively [5]. An analysis conducted among individuals aged 12–59 in China [6], along with a review of 24 studies [7], revealed that the public expressed concerns about the risk of family members contracting the virus. According to the 2021 Korean Community Health Survey [8], 60.2% of respondents expressed fear of contracting the virus, and 70.2% were worried about its economic impact. In particular, the Korean National Mental Health Survey [9] further indicates that concerns and fears about COVID-19 were highest among those in their 30 s, followed by those in their 40 s, 50 s, teens, and 60 s, in that order. Furthermore, the fear of infection profoundly affected mental health to an extent similar to the immediate threat posed by the virus. Such fears stemmed from the novelty, unpredictability, and perceived lethality of the virus and its potential impacts on personal safety, the welfare of loved ones, and economic situation [9,10]. These worries may have contributed to mental health disorders such as anxiety and depression [10].

Meanwhile, previous studies have indicated that individual characteristics such as gender, income, educational background, physical activity level, and self-perceived health status are related to COVID-19 fear and infection-prevention behaviors [11,12,13,14,15,16,17,18]. Furthermore, research on adolescents [11] and adults [12] demonstrates that physical activity levels significantly predict COVID-19-related anxieties and that individuals who perceive their health favorably are less concerned about COVID-19 than those with negative perceptions of their personal health [13,14]. Individuals with higher income levels tend to engage more frequently in infection-prevention behaviors than those living in low-income households [15,16]. Those with a college education or higher are significantly more likely to engage in infection-prevention behaviors compared to those with lower educational attainment [17]. Females reported wearing masks, washing their hands, and maintaining social distance from others more frequently than males during the COVID-19 pandemic [18].

Although the relationship among individual characteristics, COVID-19 fears, and preventive behaviors are evident, there remains a substantial need for comprehensive research in South Korea, particularly within domestic settings. This study aims to address this gap by analyzing survey data from 228,932 individuals (weighted to 43,583,798.71), conducted by the Korea Disease Control and Prevention Agency [8].

### 1.2. Theoretical Review

#### 1.2.1. Respiratory Diseases

Since the 2000s, several notable respiratory viruses have emerged, including SARS (SARS-CoV), H1N1 influenza, MERS (MERS-CoV), and COVID-19 [19,20]. SARS originated in China in 2002 and quickly spread globally, resulting in 8096 confirmed cases worldwide, including 4 in South Korea. H1N1 influenza, which first broke out in San Diego, USA, in March 2009, caused more extensive damage than SARS, with 1,632,258 confirmed cases globally and over 700,000 cases in South Korea. MERS began in Saudi Arabia in 2012 and predominantly affected the Middle East and Asia, resulting in 1288 confirmed cases worldwide and 186 cases in South Korea. The most recent respiratory virus, COVID-19, declared a pandemic, first emerged in Wuhan, Hubei Province, China, in December 2019. From 5 January 2020 to 19 May 2024, there were 775,522,404 confirmed cases and 7,049,617 deaths reported globally. During the same period, South Korea reported 34,572,554 confirmed cases and 35,605 deaths. As evidenced, respiratory infectious diseases have not been eradicated and continue to reemerge. Vaccines and antiviral treatments like Tamiflu play crucial roles in managing these infections [21,22]. However, preventive behaviors such as handwashing and mask-wearing have been scientifically proven to significantly curb the spread of infections and reduce fatalities [23,24]. Therefore, understanding how individual characteristics influence infection-prevention behaviors can aid in developing effective strategies for managing future respiratory disease outbreaks.

Meanwhile, in South Korea, research on the impact of COVID-19 on mental health has been continuously conducted [25,26,27,28,29,30,31,32]. However, many studies have focused on stress related to COVID-19 [25,26,27,28]. Although research on anxiety related to COVID-19 has been carried out, these studies are often limited to local data or targeted at specific age groups or genders [29,30,31,32]. Therefore, there is a need for research analyzing the relationship between COVID-19 and anxiety based on data that can represent the South Korean population.

#### 1.2.2. The Effect of Gender on COVID-19-Related Fears and Infection Prevention Behaviors

Previous studies have demonstrated that factors such as gender, economic activity, and occupation significantly influence concerns about COVID-19 and engagement in preventive behaviors [15,16,17,18,33]. Bronfman et al. [18] found that women are significantly more likely than men to engage in infection-prevention behaviors, such as wearing masks, washing hands upon returning home, and maintaining social distance. Women also exhibited a higher tendency to avoid going out and attending social events compared to men. The authors suggest that worry and fear about COVID-19 are crucial drivers of these infection-preventive behaviors. Differences in vaccine willingness have also been observed based on gender and educational background [33]. For instance, in France, Germany, Russia, and Sweden, women were significantly more likely to accept a vaccine than men. Moreover, previous studies [34,35,36,37,38] indicate that women experience higher levels of worry and fear about the risk of infection and the spread of respiratory infectious diseases.

#### 1.2.3. The Effect of Income and Educational Background on COVID-19 Infection Prevention Behaviors

Research by Chen and Chen [15], which analyzed 1591 people aged 16 to 71 in China, suggests that individuals with higher income levels tend to engage more frequently in infection-prevention behaviors and exhibit more positive attitudes, greater knowledge, and better information appraisal. Leung et al. [17] analyzed 1115 Chinese adults aged over 18 and found people with higher education levels adopted more thorough precautionary measures to prevent infection during the SARS outbreak in Hong Kong. Individuals with a college education or higher are significantly more likely to engage in infection-prevention behaviors compared to those with lower educational attainment. Conversely, Raifman and Raifman [16] report that individuals living in low-income households face a higher risk of contracting COVID-19 compared to their higher-income counterparts.

#### 1.2.4. The Effect of Physical Activity on COVID-19-Related Fears

A clear link has been established between physical activity and fears related to COVID-19. A study involving adolescents [11] found that physical activity levels significantly influenced COVID-19-associated anxieties, a finding was supported by a study on adults [12]. Additionally, a cross-sectional study by Maugeri et al. [39] showed that individuals who sustained physical activity levels during the COVID-19 lockdown experienced lower anxiety and fear about the pandemic than those who reduced their physical activity levels.

#### 1.2.5. The Relationship between Self-Perceived Health Status and COVID-19-Related Fears

Self-perceived health status has been shown to be inversely associated with COVID-19-related anxieties [13,14]. According to a recent study [13,14], individuals who perceive their health positively are less fearful of COVID-19 than those who view their health negatively. Moreover, recent evidence suggests that self-perceived health status might alleviate negative psychological impacts linked to COVID-19 [13,14,40].

#### 1.2.6. The Relationship between Subjective Health and Physical Activity

Maintaining an adequate level of physical activity is crucial for both physical and mental health [41]. Physical activities encompass structured exercises and sports, as well as walking or climbing stairs [41]. These activities enhance measurable health indicators, including self-perceived health status [42], which is a significant factor in psychological healthcare [43]. Research on adults and seniors has demonstrated that physical activity enhances subjective perception of health [42]. Elderly individuals who regularly engage in physical activity report significantly better self-perceived health statuses than those who are inactive [42]. Physical activity can improve self-reported health status through several mechanisms, such as increasing physical capabilities, reducing chronic disease symptoms, enhancing mood, elevating self-esteem, and promoting social interaction [42,43,44,45,46,47,48]. Furthermore, in longitudinal studies, physically active adults reported better self-assessed health than their inactive counterparts [48].

#### 1.2.7. The Effect of COVID-19 on Lifestyle Changes

COVID-19 has been found to impact lifestyle factors such as smoking and alcohol consumption [49,50,51,52]. During the lockdown, changes in tobacco consumption were observed among current smokers, with 26.7% reporting an increase, 18.6% a decrease, and 54.7% maintaining their usual consumption levels. Similarly, among those who drink alcohol, 10.7% reported consuming more, 24.4% noted a reduction, and 64.8% experienced no change in their drinking habits [49].

Based on the theoretical review above, this study was undertaken to analyze the individual characteristics (including gender, physical activity level, self-perceived health status, engagement in economic activity, occupation, smoking, and drinking) associated with fears and infection-prevention behaviors regarding respiratory infectious disease among South Korean adults during COVID-19.

## 2. Materials and Methods

### 2.1. Samples

This study used data from the 2021 Community Health Survey conducted by the Korea Disease Control and Prevention Agency [8]. The survey was conducted based on the Regional Public Health Act in Korea [8]. The target population consisted of adults aged 19 and older. The sampling frame was created by linking the resident registration population data from the Ministry of the Interior and Safety of Korea with housing data from the Ministry of Land, Infrastructure and Transport of Korea, providing a comprehensive list of the entire population. The first stage of sampling involved selecting locations proportionally to the number of households by housing type within each town/village ensuring that the probability of selection was proportional to the household size. The second stage of sampling entailed identifying the number of households in the selected town/villages and selecting households using a systematic sampling method.

### 2.2. Weights

Since the sample for the 2021 Community Health Survey was extracted under a complex sampling design rather than a simple random sampling, the weights, stratification variables, and cluster variables must be considered when estimating means and variances [8]. The weight factors were calculated and distributed by the Korea Disease Control and Prevention Agency [8], and this study utilized these weight factors accordingly. The Korea Disease Control and Prevention Agency [8] calculated the weights as follows. The weights were divided into household weights and individual weights. Household weights were calculated by considering the household sampling rate, the rate of eligible households, and the household proportions by housing type. The individual weight is a household weight adjusted for the individual response rate.

### 2.3. Procedure

**Figure 1:**
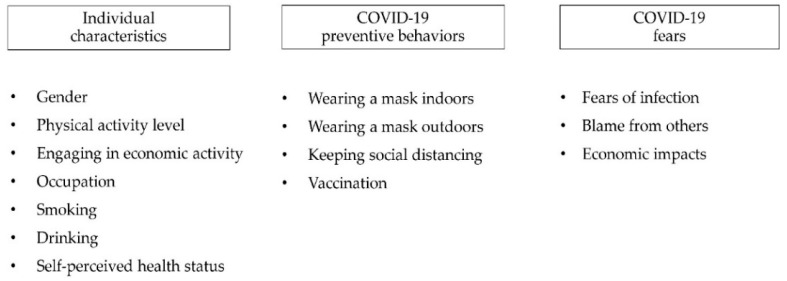
The analyzed factors of the study.

The survey period was from 16 August 2021 to 31 October 2021. Trained surveyors conducted one-on-one interviews (electronic surveys) by visiting the households selected for the sample. The data collected using tablet PCs were transmitted in real-time to a central server. The survey gathered responses from 229,242 adults aged 19 and older. However, this study excluded 310 surveys from the analysis due to non-responses or unclear answers among the analyzed factors of the study (Figure 1), resulting in a final sample size of 228,932. Applying the sampling weight factors calculated and distributed by the Korea Disease Control and Prevention Agency [8], the interpreted data represented 43,583,798.71 individuals, as indicated in Table 1. The table also includes unweighted and weighted frequencies and percentages.

### 2.4. Instruments

The 2021 Community Health Survey [8] posed questions that addressed gender, physical activity levels, economic activity, occupation, vaccination status, smoking, drinking, self-perceived health status, COVID-19-related fears and COVID-19 infection-prevention behaviors. Physical activity levels were assessed using three questions targeting high-intensity (In the past week, on how many days did you engage in vigorous physical activities for at least 10 min that made you feel much more fatigued or out of breath than usual?), moderate (In the past week, on how many days did you engage in moderate physical activities for at least 10 min that made you feel slightly more fatigued or slightly out of breath than usual?), and low-intensity (In the past week, on how many days did you walk for at least 10 min at a time?) activity levels. Each question was scored on a 7-point scale (1 = 1 day, 2 = 2 days, 3 = 3 days, 4 = 4 days, 5 = 5 days, 6 = 6 days, 7 = 7 days), and the average value was used to determine physical activity levels. A higher score indicates a higher level of physical activity. Respondents were subsequently allocated to low or high-exercise groups about the median value.

The measure of economic activity asked whether the respondent was currently engaged in economic activity, with the answer options being “yes” or “no”. The occupation categories consist of 10 groups, including manager, professional, clerical worker, service worker, sales worker, agricultural, fishery worker, technician, plant worker, elementary worker, and military personnel.

The question regarding vaccination status asked whether the respondent had received a COVID-19 vaccination up to that point, with responses categorized as either “Yes” or “No”. Questions about drinking and smoking asked respondents about changes in the frequency of their drinking and smoking habits after COVID-19, with the response options being “decreased”, “stayed the same” and “increased.

Self-perceived health status was determined using responses to a single question (How would you rate your overall health?) on overall self-perceived health status, rated on a 5-point scale (1 = very good, 2 = good, 3 = average, 4 = poor, 5 = very poor). After applying inverse coding, a higher score indicates a higher level of self-perceived health status.

COVID-19-related fears were assessed using three questions that addressed fears of infection (I am worried about getting infected with COVID-19), blame from others (I am worried that if I get infected with COVID-19, I might be blamed or face negative reactions from those around me), and economic impacts (I am worried that the COVID-19 pandemic may cause economic harm to me and my family, including losing jobs or facing difficulties in finding employment). These questions were evaluated using a 5-point scale (1 = strongly agree, 2 = agree, 3 = neutral, 4 = disagree, 5 = strongly disagree). After applying inverse coding, a higher score indicates a greater level of fear. COVID-19 preventive behaviors were assessed using three questions asking respondents whether they adhered to indoor mask-wearing, outdoor mask-wearing, and social distancing over the past week. The responses were categorized into “highly comply”, “comply” and “never”. The analyzed factors of the study are presented in Figure 1.

### 2.5. Data Analysis

Weighting methods are essential when analyzing raw data from sources, such as the Community Health Survey of Korea, to ensure sample data accurately reflect entire populations [53]. In this study, we used a complex sample design, incorporating weight, stratification, and cluster variables. The analyses included a complex sample design chi-square test, complex sample design *t*-test, and complex sample design simple regression analysis, all conducted using SPSS 20.0.

## 3. Results

### 3.1. Differences According to Gender

The complex sample design chi-square analysis results showed that, compared to the period before COVID-19, the frequency of smoking and drinking decreased for both men and women. Specifically, the analysis revealed that the reduction in smoking and drinking was more pronounced in men than in women. However, statistical significance was observed only in the change in drinking. In addition, compared to the period before COVID-19, physical activity levels decreased for both men and women, with the reduction being more pronounced in women than in men (Table 2).

The results of the complex sample design *t*-test indicated that men have a statistically significantly higher self-perceived health status compared to women (Table 3).

The results of the complex sample design t-test indicated that women have statistically significantly higher COVID-19-related fears including fear of infection, fear of blame from others, and fear of economic impacts compared to men (Table 4).

The results of the complex sample design of the t-test indicated that women have statistically significantly higher levels of infection-prevention behaviors including vaccination, wearing a mask and social distancing compared to men (Table 5).

### 3.2. Differences According to Economic Activity

The complex sample design chi-square analysis results showed that there is a difference in vaccination status, wearing a mask outdoors, and social distancing depending on engagement in economic activity. People who are engaged in economic activities showed a higher vaccination rate compared to those who are not. In terms of wearing a mask outdoors, it was found that people engaged in economic activities had a lower rate of “highly comply” compared to those who were not. However, when combining “highly comply” and “comply”, the rate was found to be identical at 99.7% for both groups. In terms of social distancing, people engaged in economic activities had higher rates of “highly comply” and “comply” compared to those who were not (Table 6).

The complex sample design chi-square test revealed differences in vaccination rates by occupation. The occupation with the highest vaccination rate was found to be military personnel, while sales workers had the lowest vaccination rate. However, all occupational groups showed a high vaccination rate of over 70%. Similarly, in terms of wearing a mask both indoors and outdoors, and practicing social distancing, military personnel exhibited the highest rate of “highly comply” (Table 7).

### 3.3. Differences According to Physical Activity Level

Complex sample *t*-test analysis revealed that the low-exercise group had a lower score for self-perceived health status than the high-exercise group (Table 8).

In terms of COVID-19-related fear, the high-exercise group had a lower level of COVID-19-related fears, including fears of infection, blame from others, and economic impact, than the low-exercise group. All differences were statistically significant (Table 9).

### 3.4. Differences According to Self-Perceived Health Status

Complex sample design simple regression analysis showed that self-perceived health status was inversely and significantly related to COVID-19-related fears including infection, blame from others, and economic impacts (Table 10).

## 4. Discussion

This study aimed to identify the individual characteristics associated with COVID-19 fears and infection-prevention behaviors among South Koreans. First, this study found a statistically significant difference in COVID-19-related fears and infection-prevention behaviors according to gender. Our findings align with previous research [18,33,34,35,36,37,38]. For instance, as compared to males, females showed significantly higher levels of COVID-19-related fears, including concerns about infection, blame from others, and economic impacts. Studies of similar respiratory infectious diseases, such as SARS-CoV-2, have also indicated that women experience higher levels of worry about the risk of infection and the spread of disease compared to men [34,35,36,37,38]. In addition, this study found that women were significantly more likely than men to participate in infection-prevention behaviors, including wearing masks, practicing social distancing, and getting vaccinated. Bronfman, Repetto, Cordón, and Castañeda [18] also observed that females more often reported wearing masks, washing their hands, keeping social distancing, and abstaining from attending social events. Previous studies [36] propose that female’s prominent caregiving roles contribute to their elevated levels of concern. Moreover, this increased anxiety among women is associated with the fear of transmitting infections to those close to them, which further underscores their proactive involvement in preventive behaviors [24] also suggested that worry and fear about COVID-19 play a crucial role in driving these infection preventive behaviors. In terms of vaccination, this study supports Lazarus et al. [33]’s finding, which indicates females in France, Germany, Russia, and Sweden were significantly more likely to accept a vaccination compared to males. Since Lazarus et al. [33]’s study also found that age might affect the willingness to accept vaccination, diverse follow-up studies are necessary to identify an individual’s vaccination willingness by region, gender, and age.

Secondly, this study found that a positive self-perceived health status is associated with less COVID-19-related fear, which aligns with previous studies [13,14,54,55]. According to Goodwin et al.’s study [54], a correlation exists between diminished self-perceived health status and increased anxiety about health, which can exacerbate fears associated with infectious diseases. Rubin et al. [55] found that the perception of subjective health status may influence fear of health threats, such as infectious diseases. Individuals who regard their health as suboptimal might perceive themselves as more susceptible to illnesses and, consequently, experience higher levels of fear. Research conducted during the H1N1 influenza pandemic demonstrated that individuals with lower self-perceived health status reported greater fear of infection [44]. Szabo, Ábel, and Boros [13] examined whether COVID-19-related fears differed among groups with high, average, or low self-perceived health status levels. Their results showed that individuals with average or low self-perceived health status expressed greater fears about COVID-19 and were more pessimistic regarding the termination of the pandemic compared to those with high self-perceived health status [13]. According to a study performed on adults 18 years and older, with an average age of 45.16 years [14], self-perceived health status was significantly and inversely correlated with COVID-19-related fear.

Thirdly, COVID-19-related fears are inversely related to physical activity levels, which concurs with findings from prior research [11,12,39,55]. The latest studies [11,12,39,56] have addressed the relationship between COVID-19-related mental health and physical activity. According to a study in 2020 by Matias et al. [56], engagement in physical activities such as yoga and tai chi during the COVID-19 pandemic positively influenced mental health, significantly reducing COVID-19-associated stress and anxiety. A cross-sectional study conducted in 2020 showed that individuals who maintained their physical activity levels during the COVID-19 lockdown experienced lower levels of anxiety and pandemic-related fear than those who decreased their physical activity levels [39]. In 2020, Alsalhe et al. [12] concluded that physical activity levels were inversely related to COVID-19 fears in a study of adults aged 18 and older. Furthermore, a recent study conducted in 2021 found that higher physical activity levels were significantly associated with lower COVID-19 fears [11]. This study was performed on German adolescents. Although we focused on adults, our findings are similar to those for adolescents.

Lastly, levels of physical activity are associated with increased self-perceived health status, which aligns with previous studies [44,45,48,57,58]. A prior study conducted in 2011 analyzed college students and reported that those perceiving their health status as “very healthy” had the highest proportion of individuals who exercised regularly [44]. Furthermore, a study involving 12 women in their 20s who engaged in trekking exercises twice a week for 10 weeks reported a significant increase in self-perceived health status levels [45]. According to Bize, Johnson, and Plotnikoff [47], physical activities can improve physical capabilities, alleviate the symptoms of chronic conditions, boost mood, increase self-esteem, and foster social connections. Moreover, they emphasized that regular physical activity could lead to a heightened sense of control over one’s health, which may, in turn, positively influence self-perceived health status. A study conducted in 2001 also indicated that adults aged 20 and older who regularly participated in exercise had higher self-perceived health status levels than those who did not [57]. According to Kim [58], engaging in sports for leisure purposes positively influences self-perceived health status. This study included a diverse range of ages, from adolescents to adults over 60.

Meanwhile, this study has the following limitations. First, the subjects of this study were limited to adults aged 19 and older. Second, it did not analyze differences based on certain demographic variables such as age and residential area (urban or rural). Third, it did not include information on whether the subjects were actually infected with COVID-19. Fourth, comparing this study’s results with global findings requires caution, especially without a clear direct link between them. Fifth, this study used a complex sample design focused on population-level inference. Therefore, when interpreting the results, it is important to consider findings from similar studies that used simple random sampling.

## 5. Conclusions

In summary, this study aims to update the understanding of individual characteristics affecting fears and infection-prevention behaviors related to respiratory infectious diseases. This study holds several significant contributions. The subjects are Korean, and the data are based on a national survey, providing valuable insights specific to this population. The results of this study offer the potential to integrate and analyze research data from different regions and races worldwide. Additionally, as COVID-19 is the most recent global pandemic, this study builds on previous research on SARS and other past pandemics, providing foundational data for updated research. Furthermore, this study demonstrated that maintaining sufficient physical activity positively influences self-perceived health and reduces anxieties related to COVID-19 infection and fatalities. This emphasizes the importance of identifying strategies that effectively sustain physical activities and exercise while adhering to infection-prevention measures during pandemics.

## Figures and Tables

**Table 1:** Demographic information (*N* = 43,583,798.71).

Type		Weighted		Unweighted
		*N*	%	*N*
Male		21,607,261.52	49.6%	104,352
Female		21,976,537.19	50.4%	124,580
Age	19–29	7,250,736.91	16.6%	24,679
	30–39	6,760,819.29	15.5%	25,716
	40–49	8,202,946.71	18.8%	35,509
	50–59	8,586,211.27	19.7%	43,027
	60–69	6,728,597.88	15.4%	47,457
	70–79	4,112,996.15	9.4%	33,981
	80 or older	1,941,490.47	4.5%	18,563
Educational Background *	Elementary school	3,727,487.24	8.6%	35,905
	Middle school	3,334,616.72	7.7%	24,744
	High school	12,663,486.19	29.1%	66,300
	2-year/3-year college	6,092,725.57	14.0%	25,964
	4-year university	14,322,020.67	32.9%	54,635
	Graduate school or higher	2,413,120.98	5.5%	9085
	No formal education	987,309.53	2.3%	11,944
	Others	21,213.69	0.0%	222
Engagement in Economic Activity *	Yes	27,732,897.89	63.6%	143,344
	No	15,848,981.18	36.4%	85,580
Physical Activity Level	Low	20,845,572.90	47.8%	115,323
	High	22,738,225.80	52.2%	113,609

* Respondents who refused to answer or answered “don’t know” were excluded.

**Table 2:** Differences of change in smoking, drinking and physical activity level according to gender after the COVID-19 pandemic.

Gender	Smoking			Drinking			Physical Activity Level		
	Decreased	Same	Increased	Decreased	Same	Increased	Decreased	Same	Increased
Male	20.1%	68.9%	11.0%	47.0%	45.0%	8.0%	42.8%	48.8%	8.4%
Female	19.8%	67.9%	12.3%	42.3%	48.8%	8.9%	49.5%	41.8%	8.7%
Total	20.1%	68.8%	11.1%	45.0%	46.6%	8.4%	46.2%	45.3%	8.6%
χ^2^	2.927			96.684 ***			343.179 ***		

*** = *p* < 0.001.

**Table 3:** Differences in self-perceived health status by gender.

Variable	Parameter	Weighted			*t*
		*N*	*M*	Standard Error	
Gender	Male	21,607,261.52	3.48	0.003	45.522 ***
	Female	21,976,537.19	3.29	0.003	

*** = *p* < 0.001.

**Table 4:** Differences in COVID-19-related fears by gender.

Fear of Infection					
Variable	Parameter	*N*	*M*	Standard Error	*t*
Gender	Male	21,607,261.52	3.54	0.004	−64.541 ***
	Female	21,976,537.19	3.89	0.003	
**Fear of Blame from Others**					
**Variable**	**Parameter**	***N***	***M***	**Standard Error**	***t***
Gender	Male	21,607,261.52	3.70	0.004	−47.021 ***
	Female	21,976,537.19	3.97	0.004	
**Fear of Economic Impacts**					
**Variable**	**Parameter**	***N***	***M***	**Standard Error**	***t***
Gender	Male	21,607,261.52	3.82	0.004	−28.382 ***
	Female	21,976,537.19	3.98	0.004	

*** = *p* < 0.001.

**Table 5:** Differences in compliance with infection-prevention behaviors according to gender.

Gender	Vaccination		Wearing a Mask Indoors			Wearing a Mask Outdoors			Social Distancing		
	Yes	No	Highly Comply	Comply	Never	Highly Comply	Comply	Never	Highly Comply	Comply	Never
Male	76.1%	23.9%	90.9%	8.9%	0.2%	89.9%	9.6%	0.4%	71.6%	25.8%	2.7%
Female	78.5%	21.5%	93.1%	6.8%	0.1%	92.4%	7.4%	0.2%	74.1%	23.6%	2.3%
Total	77.3%	22.7%	92.0%	7.8%	0.1%	91.2%	8.5%	0.3%	72.9%	24.7%	2.5%
χ^2^	141.747 ***		113.783 ***			179.612 ***			57.620 ***		

*** = *p* < 0.001.

**Table 6:** Differences of compliance with infection-prevention behaviors according to engagement in economic activity.

Engagement in Economic Activity	Vaccination		Wearing a Mask Indoors			Wearing a Mask Outdoors			Keeping Social Distancing		
	Yes	No	Highly Comply	Comply	Never	Highly Comply	Comply	Never	Highly Comply	Comply	Never
Yes	77.7	22.3	92.0	7.9	0.1	91.1	8.6	0.3	72.9	24.7	2.4
No	76.7	23.3	92.1	7.8	0.2	91.6	8.1	0.3	72.6	24.6	2.9
Total	77.3	22.7	92.0	7.8	0.1	91.2	8.5	0.3	72.9	24.7	2.5
χ^2^	17.427 ***		1.631			4.777 **			10.226 ***		

*** = *p* < 0.001, ** = *p* < 0.01.

**Table 7:** Differences in compliance with infection-prevention behaviors according to occupation.

Occupation	Vaccination		Wearing a Mask Indoors			Wearing a Mask Outdoors			Keeping Social Distancing		
	Yes	No	Highly Comply	Comply	Never	Highly Comply	Comply	Never	Highly Comply	Comply	Never
Manager	83.0%	17.0%	92.6%	7.3%	0.1%	92.2%	7.6%	0.3%	75.2%	22.0%	2.8%
Professional	79.3%	20.7%	93.7%	6.2%	0.1%	93.2%	6.6%	0.2%	74.6%	22.5%	2.9%
Clerical worker	73.9%	26.1%	92.8%	7.1%	0.1%	92.5%	7.2%	0.2%	73.3%	24.3%	2.4%
Service worker	79.0%	21.0%	92.7%	7.1%	0.1%	92.2%	7.6%	0.2%	74.0%	23.5%	2.4%
Sales worker	73.0%	27.0%	91.6%	8.1%%	0.3%	91.0%	8.7%	0.3%	70.1%	26.6%	3.3%
Agricultural, fishery worker	88.8%	11.2%	88.1%	11.6%	0.3%	83.5%	15.2%	1.3%	67.7%	29.7%	2.6%
Technician	73.4%	26.6%	90.8%	9.0%	0.2%	89.7%	9.7%	0.6%	69.3%	27.9%	2.8%
Plant worker	75.3%	24.7%	91.4%	8.4%	0.1%	90.6%	9.1%	0.3%	69.1%	27.6%	3.3%
Elementary worker	81.5%	18.5%	91.1%	8.8%	0.2%	89.8%	9.9%	0.4%	69.7%	27.6%	2.7%
Military personnel	96.4%	3.6%	94.5%	5.5%	0.0%	94.4%	5.5%	0.1%	80.2%	18.2%	1.6%
Total	77.7%	22.3%	92.2%	7.7%	0.0%	91.3%	8.4%	0.3%	72.1%	25.2%	2.8%
χ^2^	98.530 ***		13.008 ***			34.496 ***			15.282 ***		

*** = *p* < 0.001.

**Table 8:** Differences in self-perceived health status by physical activity level.

Variable	Parameter	Weighted			*t*
		*N*	*M*	Standard Error	
Physical Activity	Low	20,845,572.91	3.2698	0.00346	49.725 ***
	High	22,738,225.80	3.4995	0.00330	

*** = *p* < 0.001.

**Table 9:** Differences in COVID-19-related fears by physical activity level.

Fear of Infection					
Variable	Parameter	*N*	*M*	Standard Error	*t*
Physical Activity	Low	20,845,572.91	3.7548	0.00419	11.515 ***
	High	22,738,225.80	3.6878	0.00444	
**Fear of Blame from Others**					
**Variable**	**Parameter**	***N***	***M***	**Standard Error**	***t***
Physical Activity	Low	20,845,572.91	3.8577	0.00443	4.871 ***
	High	22,738,225.80	3.8281	0.00462	
**Fear of Economic Impacts**					
**Variable**	**Parameter**	***N***	***M***	**Standard Error**	***t***
Physical Activity	Low	20,845,572.91	3.9343	0.00468	8.413 ***
	High	22,738,225.80	3.8814	0.00487	

*** = *p* < 0.001.

**Table 10:** The effect of self-perceived health status on COVID-19-related fears.

COVID-19 Infection According to Subjective Health					
Dependent Variable	Independent Variable	Estimate	Standard Error	*t*	*F*
Fear of infection	Intercept	4.077	0.012	326.441 ***	830.699 ***
	Subjective health	−0.105	0.004	−28.822 ***	
**COVID-19 Blame according to Subjective Health**					
**Dependent Variable**	**Independent Variable**	**Estimate**	**Standard Error**	***t***	***F***
Fear of blame from others	Intercept	4.084	0.013	320.579 ***	359.296 ***
	Subjective health	−0.071	0.004	−18.955 ***	
**COVID-19 Economic according to Subjective Health**					
**Dependent Variable**	**Independent Variable**	**Estimate**	**Standard Error**	***t***	***F***
Fear of economic impacts	Intercept	4.272	0.013	336.073 ***	825.310 ***
	Subjective health	−0.108	0.004	−28.728 ***	

*** = *p* < 0.001.

## Data Availability

Restrictions apply to the availability of these data. Data were obtained from [Korea Disease Control and Prevention Agency] and are available [at https://chs.kdca.go.kr/chs/index.do (accessed on 20 September 2024)] with the permission of [Korea Disease Control and Prevention Agency].
